# Averaging of Viral Envelope Glycoprotein Spikes from Electron Cryotomography Reconstructions using Jsubtomo

**DOI:** 10.3791/51714

**Published:** 2014-10-21

**Authors:** Juha T. Huiskonen, Marie-Laure Parsy, Sai Li, David Bitto, Max Renner, Thomas A. Bowden

**Affiliations:** ^1^Oxford Particle Imaging Centre, Division of Structural Biology, Wellcome Trust Centre for Human Genetics, University of Oxford

**Keywords:** Immunology, Issue 92, electron cryo-microscopy, cryo-electron microscopy, electron cryo-tomography, cryo-electron tomography, glycoprotein spike, enveloped virus, membrane virus, structure, subtomogram, averaging

## Abstract

Enveloped viruses utilize membrane glycoproteins on their surface to mediate entry into host cells. Three-dimensional structural analysis of these glycoprotein ‘spikes’ is often technically challenging but important for understanding viral pathogenesis and in drug design. Here, a protocol is presented for viral spike structure determination through computational averaging of electron cryo-tomography data. Electron cryo-tomography is a technique in electron microscopy used to derive three-dimensional tomographic volume reconstructions, or tomograms, of pleomorphic biological specimens such as membrane viruses in a near-native, frozen-hydrated state. These tomograms reveal structures of interest in three dimensions, albeit at low resolution. Computational averaging of sub-volumes, or sub-tomograms, is necessary to obtain higher resolution detail of repeating structural motifs, such as viral glycoprotein spikes. A detailed computational approach for aligning and averaging sub-tomograms using the Jsubtomo software package is outlined. This approach enables visualization of the structure of viral glycoprotein spikes to a resolution in the range of 20-40 Å and study of the study of higher order spike-to-spike interactions on the virion membrane. Typical results are presented for Bunyamwera virus, an enveloped virus from the family *Bunyaviridae*. This family is a structurally diverse group of pathogens posing a threat to human and animal health.

**Figure:**
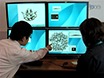


## Introduction

Electron cryo-tomography is an electron cryo-microscopy imaging technique allowing the calculation of a three-dimensional (3D) reconstruction of complex biological specimens. Suitable specimens range from purified macromolecular complexes^1^, filaments^2^, coated vesicles^3^, and pleomorphic membrane viruses^4^ to whole prokaryotic cells^5^ and even thin areas of whole eukaryotic cells^6^. Following the data collection of a tilt-series, 3D tomographic volumes, or tomograms, may be calculated using several established software packages, including Bsoft^7^ and IMOD^8^.

Two aspects inherent to the study of biological specimens by electron cryo-tomography limit the biological interpretation of the corresponding tomographic volumes. First, due to the limited electron dose that can be applied to biological materials before introducing significant radiation damage, signal-to-noise ratios in tomographic data are typically very low. Second, as a result of limited sample tilt geometry during data collection, some views of the object remain absent, leading to a so-called ‘missing wedge’ artifact in the tomographic volume. However, both of these limitations can be overcome if the tomographic volume contains repeating identical structures, such as macromolecular complexes, that can be successfully averaged^9-12^.

Prior to averaging structures from tomogram reconstructions, objects of interest must be found and aligned to the same orientation. Locating such structures may be achieved by cross-correlation of a template structure in the tomographic volume using an approach often referred to as template matching^13^. The template used in this matching process can be derived from electron cryo-microscopy or electron cryo-tomography combined with 3D reconstruction, or it can be a density map simulated from an atomic structure. Several computational packages have been developed to carry out these tasks^11^.

Averaging of glycoprotein spikes of membrane viruses, such as HIV-1, has been a particularly successful approach for studying their structure^14-16^. An understanding of the structure is integral for revealing both the molecular basis of virus–host interactions and guiding antiviral and vaccine design development. While macromolecular crystallography is the technique of choice for high-resolution (usually better than 4 Å) structural analysis of individual viral glycoproteins and their complexes, the X-ray structures resulting from this method are of proteins isolated from the natural membranous environment on the virion. Thus, important details such as the higher order architecture of viral glycoproteins, in the context of the virion, remain lacking. On the other hand, electron cryo-microscopy and single particle reconstruction of entire enveloped viruses is restricted to virions with icosahedral symmetry^17,18^. Electron cryotomography combined with sub-volume alignment has thus emerged as a complementary technique allowing the study of glycoprotein spikes of irregularly shaped, pleomorphic viruses *in situ*.

We have developed software named Jsubtomo (www.opic.ox.ac.uk/jsubtomo) for the detection, alignment, and averaging of tomographic sub-volumes. Jsubtomo has been utilized in the structure determination of a number of cellular and viral structures^19-26^. Here, we outline a detailed protocol, which enables the determination viral-surface spike structures. To circumvent over-refinement of averaged structures by correlating noise, the ‘gold-standard’ refinement scheme is adopted^10,27^. Finally, strategies for visualization and interpretation of typical results are discussed.

## Protocol

A detailed protocol for the computational alignment and subsequent averaging of viral glycoprotein spikes is outlined. The protocol follows the workflow illustrated in **Figure 1** and combines an automated search for the spikes using initial template structures and a gold-standard structure refinement.

Input data for this protocol is a set of tomographic reconstructions of the virions. One tomogram contains one or more virions. Initially, a small subset of spikes is manually picked and used to average and refine two independent models. These models are used to automatically locate spikes on all of the virions. Finally, two independent refinements are run and the resulting averages are compared and combined to produce the final structure.

The refinement approach is demonstrated by using programs from the Jsubtomo package. Programs from the Bsoft package^28^ are used for general image processing tasks and molecular graphics package UCSF Chimera^29^ is used to visualize results. The names of individual programs are given in italics and file formats are denoted with uppercase filename extensions.


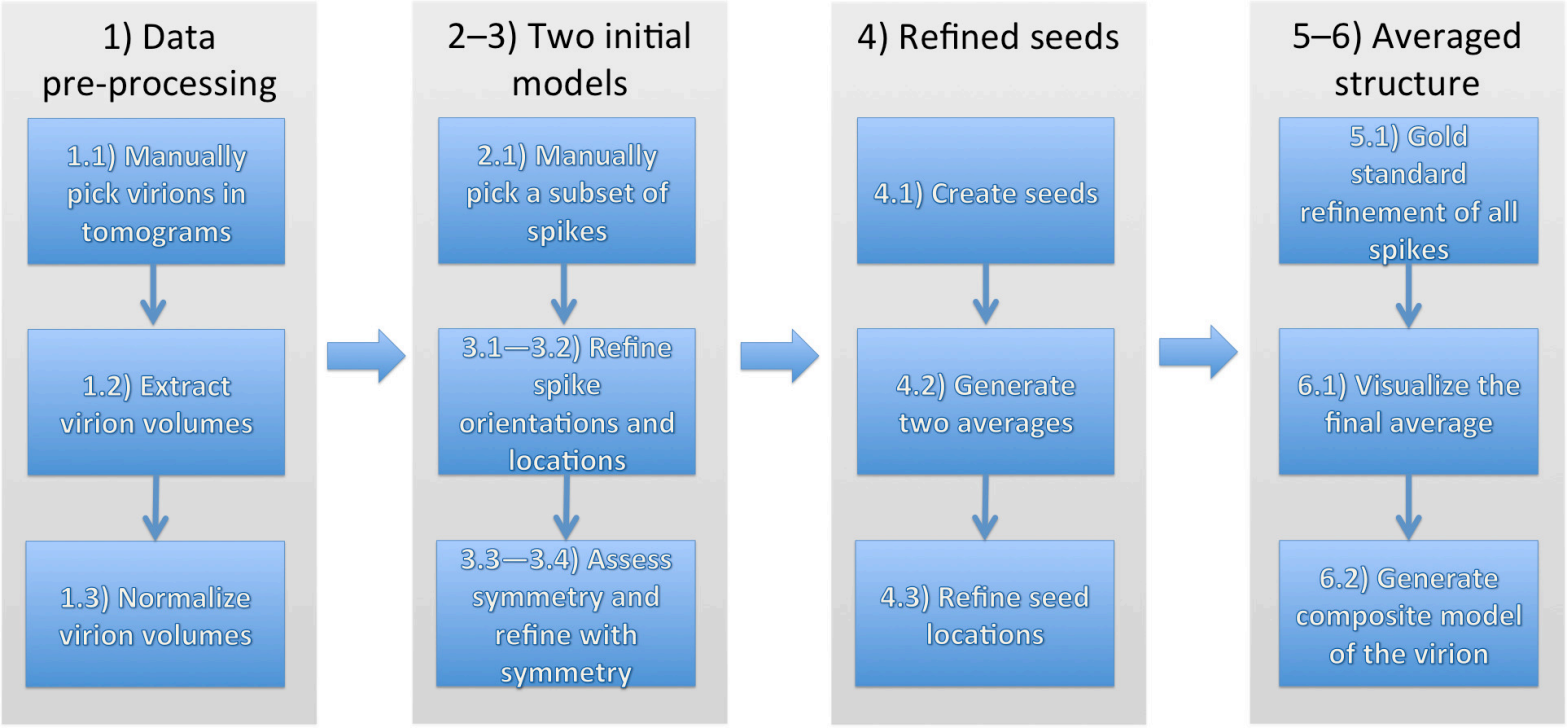
**Figure 1: General strategy for determining structures of glycoprotein spike complexes from pleomorphic membrane viruses.** The numbers correspond to different sections in the Protocol. Please click here to view a larger version of this figure.

### 1. Extracting Virus Sub-volumes from Full-size Tomograms

1. Pick virus particles manually in the tomograms.
  1. Open a tomogram file in *bshow*. Define the center coordinate of a virus particle using the particle-picking tool. Repeat this step until all virions have been processed. Save virus coordinates in a STAR file.
  2. Repeat step 1.1.1 until all tomograms have been processed. Make note of the virion diameter in pixels. NOTE: If the spikes are difficult to see in the tomograms, use *bfilter* to low-pass filter the tomograms to 80-Å resolution (parameter “-bandpass 80,10000”).
2. Run *jsubtomo.py* in extract mode (parameter “--mode extract”) to extract virion sub-volumes to individual volume files. Use the STAR files saved in step 1.1.1 as input files.
  1. Give a size (parameter “--size”) ~25% larger than the largest virion in the data set. *E.g.*, if the virion is ~200 pixels in diameter, use size 250 x 250 x 250 pixels.
  2. Output files of the virion volumes will be in MAP-format. In addition, create an accompanying STAR-file for each virion volume (parameter “--output”).
3. Normalize virion volumes in *bimg* (parameter “-rescale 0,1”).

### 2. Generation of Two Independent Initial Models

1. Pick a subset of spikes in the virion sub-volumes.
  1. Open a virion sub-volume MAP file in *bshow*. Define the center coordinate of a spike using the particle-picking tool, accessible via the Toolbox window. Repeat this step until all clearly distinct spikes have been processed. Save spike coordinates in a STAR file.
  2. If necessary, repeat step 2.1.1 for other virions until approximately 200 glycoprotein spikes have been processed. Note spike dimensions in pixels. NOTE: ~200 spikes are a rough guideline and more may be required for some applications. If possible, aim to pick spikes in different orientations. Avoid picking only top views.
2. Assign an initial view vector to the spikes by running *jviews.py*. Use the STAR files generated in step 2.1.1 as input files. NOTE: This view vector approximates the direction of the spike with reference to the virion.
  1. Use the central coordinate to assign the views for a spherical virion. For example, if the virion is centered (after step 1.2) in a box with a size of 250 x 250 x 250 pixels, the central coordinate is 125,125,125 pixels (option “--Radial 125,125,125”).
  2. To assign the views for a filamentous virion, open each virion sub-volume in *bshow* by using the STAR file defined in step 2.1.1 as the input file. Define the two end points of the filament using the filament picking tool and save the STAR file. Repeat this step until all STAR files have been updated and run *jviews.py* using the updated STAR files as input files (--option Segment).
3. Generate a real space mask using *jsubtomo_create_mask.py*. Ensure that the real space mask is of the same dimensions as will be used for the averaged structure (see 2.6.2) and is large enough to contain both the spike and a patch of the underlying membrane.
4. Generate a reciprocal space mask using *jsubtomo_create_wedgemask.py*. The reciprocal space mask is used to exclude regions in the ‘missing wedge’ region resulting from single axis tomographic data collection. Ensure that it is of the same dimensions as will be used for the averaged structure (see 2.6.2).
5. Generate a selection file (SEL file) defining virions belonging to sets “1” and “2” using *jsubtomo_evenodd.py* and the STAR files generated in step 2.2.
6. Generate two initial averages using *jsubtomo_create_averages.py*.
  1. Use the SEL file generated in step 2.5 as the input file.
  2. Give a size (parameter “--size”) at least 32 pixels larger than the largest dimension of the spike. *E.g.*, if the spike is ~40 pixels long, use size 72 x 72 x 72 pixels.
  3. Apply a high symmetry (*e.g.*, c100) on the averages (parameter “--symmetry c100”), to approximate a cylindrical average around the long-axis of the spike. Use symmetrization to reduce noise in the initial averages.
  4. Provide a unique name for the output files of the project (parameter “--suffix”).
  5. Use the real space mask generated in step 2.3 to mask away background (parameter “--mask”). Use the reciprocal space mask generated in step 2.4 to account for the loss of signal introduced by the presence of a missing wedge (parameter “--Mask”).

### 3. Gold-standard Iterative Alignment and Averaging of the Two Initial Spike Models

Iteratively align and average the two initial models generated in section 2 with *jsubtomo_iterate_gold.py*.

1. Stage I. The goal of this stage is to refine the direction of the view vector. The input file is the SEL file generated in step 2.5.
  1. Use an 8-degree angular sampling (parameter “--angles 8,8,8”).
  2. Allow 16-degree changes in the direction of the spike view vector (parameter ”--thetaphilimit 16”) but keep the angle around the spike long axis fixed (parameter “--alphalimit 0”).
  3. Allow small translational shifts (*e.g.*, 5 pixels) to account for inaccuracies in manual picking of the spikes (parameter “--shiftlimit 5”).
  4. Apply a high symmetry (*e.g.*, c100) on the averages (parameter “--symmetry c100”), to approximate a cylindrical average around the long-axis of the spike. NOTE: Symmetrization is used to reduce noise in the averages.
  5. Use the real space mask and the reciprocal space mask generated in steps 2.3 and 2.4 to mask away surrounding spikes and account for the missing wedge (parameters “--mask” and “--Mask”, respectively).
  6. Use the two MAP files generated in 2.6 (denoted by tags “even” and “odd” in the filename) as templates (parameters “--Template1” and “--Template2”, respectively).
  7. Apply a low pass filter at 50-Å resolution (parameter “--resolution”) to prevent alignment bias.
  8. Apply a bin factor of 2 to speed up the refinement (parameter “--bin 2”).
  9. Run 5 iterations of alignment and averaging (parameters “--firstiter 1 --lastiter 5”).
2. Stage II. The goal of this stage is to refine the angle around the view vector. The input file is the output SEL file from step 3.1.9.
  1. Measure the accurate dimensions of the spike by opening the low-pass filtered MAP file generated in the last iteration in step 3.1.9 (denoted by tag “_lp” in the filename) in *bshow*. Generate a new real space mask similarly to step 2.3 that defines the spike but excludes most of the membrane and neighboring spikes. NOTE: The optimal size of the mask depends on the size and features of the spike.
  2. Use 8-degree angular sampling (parameter “--angles 8,8,8”) as before.
  3. Allow 180-degree changes in the angle around the spike view vector (parameter “-- alphalimit 180”) but keep the theta and phi angles fixed (parameter “--thetaphilimit 0”).
  4. Do not allow any translational shifts (parameter “--shiftlimit 0”).
  5. Do not apply any symmetry on the averages (omit parameter “--symmetry”).
  6. Use the reciprocal space mask generated in step 2.4 to account for the missing wedge.
  7. Use the two MAP files generated in the last iteration of step 3.1.9 without symmetry (denoted by tags “even_nosym” and “odd_nosym” in the filename) as templates (parameters “--Template1” and “--Template2”, respectively).
  8. Apply a low pass filter at 50-Å resolution (parameter “--resolution”) in the first iteration to prevent alignment bias. Adjust the filter parameters in the subsequent iterations automatically (parameter “--adaptivefilter”) based on gold-standard Fourier Shell Correlation (FSC) between the two independent averages. Use 0.143-criterion (parameter “--fsccrit 0.143”). Do not allow refinement past the first zero of the contrast transfer function (CTF) (parameter “--minhires”), unless CTF correction has been applied to the tomograms.
  9. Run 5–10 iterations of alignment and averaging (*e.g.*, parameters “--firstiter 6 --lastiter 15”). Monitor the changes in the reported resolution. When no significant changes are observed, the process can be stopped.
3. Assess the degree of symmetry in the structure by examining the resulting low-pass filtered MAP file (denoted by tag “_lp” in the filename) in *chimera*. *E.g.*, if the spike is a trimeric complex, 3-fold symmetry should be evident.
4. Stage III. The goal of this stage is to refine the angle around the view vector further with correct symmetry. The input file is the output SEL file from step 3.2.9. The parameters are the same as in step 3.2 with a few exceptions:
  1. Allow appropriate changes in the angle around the spike view vector. For example, if the structure has 3-fold symmetry, allow changes of 60 degrees in the alpha angle (parameter ”--alphalimit 60”).
  2. Apply the correct symmetry (*e.g.*, c3) on the averages (parameter --symmetry c3”).
  3. Use the two MAP files generated in the last iteration in step 3.2.9 (denoted by tags “even” and “odd” in the filename) as input template files (parameters “--Template1” and --Template2).
  4. Run 5–10 iterations of alignment and averaging (*e.g.*, parameters “--firstiter 16 --lastiter 25”). Monitor the changes in the reported resolution. When no significant changes are observed, the process can be stopped.
5. Stage IV. The goal of this stage is to accurately refine all three angles simultaneously. The input file is the output SEL file from step 3.4.4. The parameters are the same as in step 3.4 with a few exceptions:
  1. Allow 8-degree changes in the angle around the spike view vector (parameter ”-- alphalimit 8”) and 8-degree changes in the direction of the spike view vector (parameter “--thetaphilimit 8”). Refine the orientations to 4-degree accuracy (parameters “--angles 8,8,8 --iterate 2”).
  2. Allow small shifts (*e.g.*, 5 pixels) to account for inaccuracies in previous alignment steps (parameter “--shiftlimit 5”).
  3. Use the two MAP files generated in the last iteration in step 3.4.4 (denoted by tags “even” and “odd” in the filename) as input template files (parameters “--Template1” and --Template2).
  4. Run 5-10 iterations of alignment and averaging (*e.g.*, parameters “--firstiter 26 --lastiter 35”). Monitor the changes in the reported resolution. When no significant changes are observed, the process can be stopped.

### 4. Generation and Alignment of Seeds to the Virus Surface for Template Matching

1. Generate seeds located evenly on the virion surface for template matching and assign an initial view vector to the seeds by running *jviews.py*. Use the STAR files generated in step 1.2.2 as input files. Generate approximately 1.5 times more seeds than the expected number of spikes. NOTE: This view vector approximates the direction of the spike closest to each seed point.
  1. To generate evenly distributed seeds on a roughly spherical virion (parameter ”--Even”), give the radius (parameter “--radius”), angular separation (*e.g.*, 20 degrees) of the seeds (parameter “--angle 20”) and the central coordinate of the virion. *E.g.*, if the virion is centered (after step 1.2) in a box with a size of 250 x 250 x 250 pixels, the central coordinate is 125,125,125 pixels (option “--origin 125,125,125”).
  2. To generate evenly distributed seeds on a filamentous particle, use the parameter “--Filament” and specify both the radius and helical symmetry parameters (rise and twist). NOTE: The helical symmetry parameters are used here only as a convenient way of defining evenly distributed seed positions and they do not need to reflect the actual ordering of the spikes on the filament.
2. Generate two independent averages of the virus surface as explained in section 2.
  1. Generate a SEL file defining virions belonging to sets “1” and “2” using *jsubtomo_evenodd.py* and the STAR files generated in step 4.1. NOTE: To ensure the independence of datasets, any previous assignment of the virions into groups “1” and “2” must stay the same.
3. Refine the position of the seeds.
  1. Follow the instructions in step 3.1 unless stated otherwise. Use the SEL file generated in step 4.2.1 as the input file.
  2. Allow the seeds to shift only along the normal to the membrane (parameter “--zshiftlimit”). Adjust the allowed amount of shift depending on how much the virions deviate from ideal spherical geometry (*e.g.*, parameter --zshiftlimit 25”).
  3. Use the two MAP files generated in step 4.2 (denoted by tags “even” and “odd” in the filename) as input template files (parameters “--Template1” and --Template2).
  4. Use a unique suffix to avoid overwriting the original input STAR files (*e.g.*, parameter “--suffix _seeds”).
  5. Use binning of 4 to speed up the calculation (parameter “--bin 4”) and a low-pass filter (*e.g.*, parameter “--resolution 50”).
4. Generate Chimera marker files (CMM) of the refined seed STAR files using *jviews.py* (parameter “--cmm”). Examine the seeds by opening the CMM files (and the associated virion MAP files) in *chimera.* Make sure that the refined seeds are aligned correctly relative to the virus membrane. NOTE: Different colors can be used to differentiate separate sets of markers (parameter “--color”). Alternatively the refined markers can be colored based on their cross correlation coefficient (*e.g.*, parameter “--fomcolor 0.1,0.3”).

### 5. Gold-standard Iterative Alignment and Averaging of the Spike Structure

Automatically locate all the spikes in the virion sub-volumes using local template matching around the refined seeds and align and average the located spikes. Use the averages generated from a subset of manually picked spikes as initial templates.

1. Perform local template matching around the seeds to average all the spikes using program *jsubtomo_iterate_gold.py*. Follow the instructions in step 3.5 unless stated otherwise.
  1. The input file is the SEL file for the refined seeds generated in step 4.3.
  2. Use a sufficiently large limit for the view vector angle. *E.g.*, if seeds were generated every 20 degrees (step 4.1), use an angle slightly larger than half of this value (parameter “--thetaphilimit 12”). Allow appropriate changes in the angle around the spike view vector.
  3. Allow seeds to shift in the plane of the membrane (parameter “--xyshiftlimit”). *E.g.*, if the seed-to-seed distance is 25 pixels, use total shift slightly more than half of this (*e.g.*, parameters “--shiftlimit 6 --xyshiflimit 10”). NOTE: Additional translational shifts may be required if the MAP files generated in step 3.5 are centered at a different distance from the virion center than the seeds.
  4. Use the two MAP files generated in step 3.5 (denoted by tags “even” and “odd” in the filename) as input template files (parameters “--Template1” and --Template2).
  5. Use the current resolution of the MAP files generated in step 3.5 (parameter --resolution).
  6. Run 5-10 iterations of template matching, alignment and averaging (*e.g.*, parameters “--firstiter 1 --lastiter 10”). Monitor the changes in the reported resolution. When no significant changes are observed, the process can be stopped.
  7. After each iteration, exclude overlapping hits. *E.g.*, if the width of the spike is 30 pixels, exclude hits that are closer than 30 pixels to other hits (parameter “--mindist 30”).
  8. Exclude hits with a low cross correlation co-efficient. For example, include the best 75% spikes for each virion (parameter “--topp 75”). NOTE: Hits with a low cross correlation co-efficient are likely to represent false positive hits.

### 6. Visualization of the Results

1. Open the refined structure of the spike in *chimera* for visualization and fitting of atomic structures.
  1. Use the low-pass filtered MAP file (denoted by tag “lp”) created in the final iteration in step 5.1.6 as the input file.
  2. Use the resolution indicated in step 5.1.6 for cross-correlation based fitting in *chimera* “Fit in Map” tool.
2. Create a composite model of the virion using *jsubtomo_create_model.py. *
  1. Use the low-pass filtered MAP file (denoted by tag “lp”) created in the final iteration in step 5.1.6 as the input MAP file to (parameter “--Template”).
  2. Use a STAR file generated in the final iteration in step 5.1.6 as the input STAR file.
  3. Use the mask file generated in step 3.2.1 to account for overlapping densities in the composite model (parameter “--mask”).
  4. Indicate the size of the virion MAP file to generate a composite model of the same size (parameter “--size”).
3. Open the composite model for visualization in *chimera*.

## Representative Results

We demonstrate the application of the sub-tomogram averaging workflow outlined above for the envelope glycoprotein complex of Bunyamwera virus (Orthobunyavirus, *Bunyaviridae*) using a previously published data set^24^. Data collection and refinement parameters are listed in **Table 1**. One representative tomogram is shown in **Figure 2**.

**Table:** 

**Parameter (unit)**	**Value**
**Data collection**	
Voltage (kV)	300
Calibrated magnification (X)	111,000
Pixel size (Å)	5.4
Estimated dose (e^–^/ Å^2^)	100
Underfocus (μm)	4.0–4.5
First CTF zero (Å)^a^	26–30
Tilt range (°)	–60–60
Tilt sampling (°)	3
**Data and refinement**	
Tomograms	11
Virions	29
Seeds per virion	106
Total number of seeds	3,074
Spikes detected	1,346
Spikes after removing overlaps	1,401
Spikes after cross-correlation based selection	1,022
Spikes included in the final average	1,022
Symmetry	C3
Angular sampling (°)	8
Resolution range used in the refinement (Å)	42–334
Final resolution estimate (Å)^b^	35

**Table 1: Bunyamwera data collection and refinement statistics.**^a^ CTF, contrast transfer function. ^b^ Calculated using Fourier shell correlation between two independently refined structures at a threshold of 0.143.


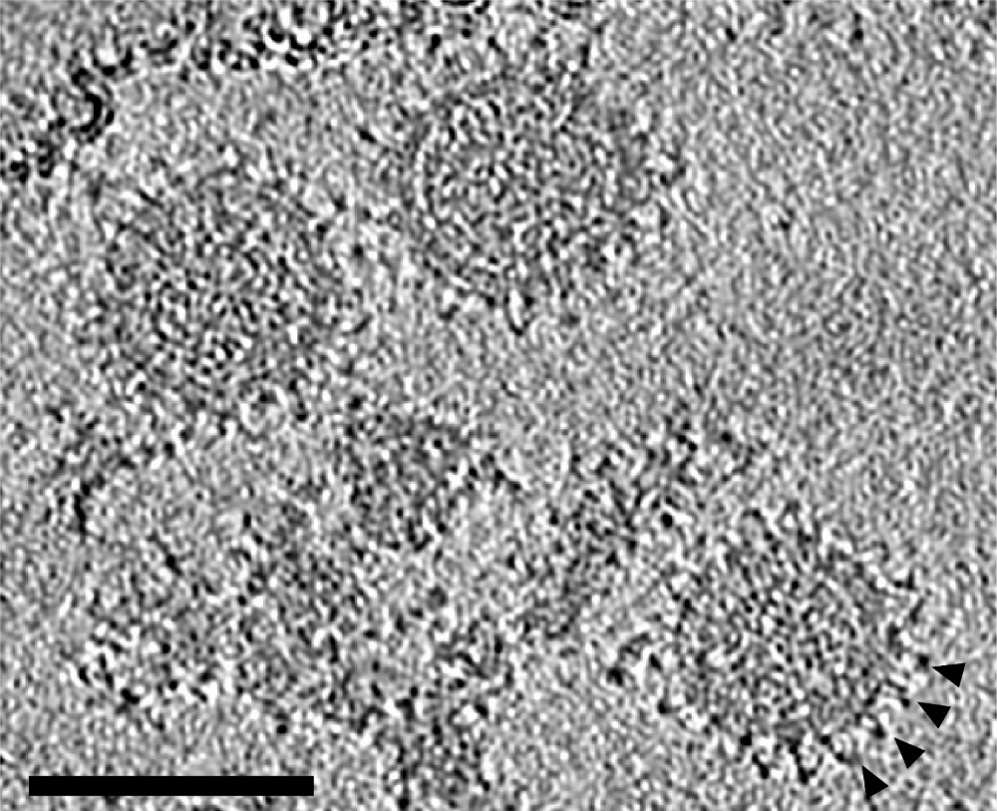
**Figure 2: Slice through a tomogram of Bunyamwera virions.** Several spike side views evident in the periphery of each virion are indicated with arrowheads. The tomogram has been low-pass filtered to 60 Å. Scale bar 100 nm.

First, we refined an initial model using 205 manually picked spikes (**Figure 3**). Three-fold symmetry of the center-most spike was evident without applying any symmetry (**Figure 3B**) and was imposed in the subsequent rounds of refinement (**Figure 3C**). For detecting all the spikes automatically on the virion surfaces, we generated 106 seeds for each virion at the radius of 43 nm and spacing of 20 degrees (**Figure 4A**) and iteratively refined their positions relative to the membrane (**Figure 4B**).


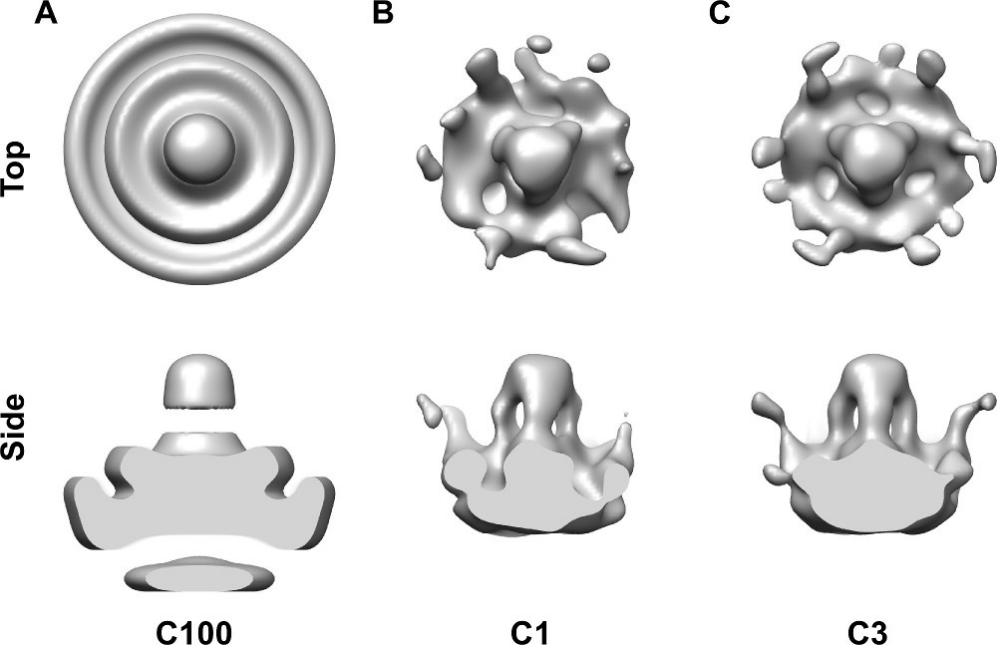
**Figure 3: Refinement of the initial template structure. (A)** Cylindrically averaged (C100) template constructed from manually defined positions of spikes. **(B)** Averaged density after five rounds of refinement without any symmetry (C1) imposed displays a spike with three-fold symmetric features. The resolution of the model is 48 Å. **(C)** Average of the spike was resolved at 41 Å after five rounds of refinement with three-fold symmetry.


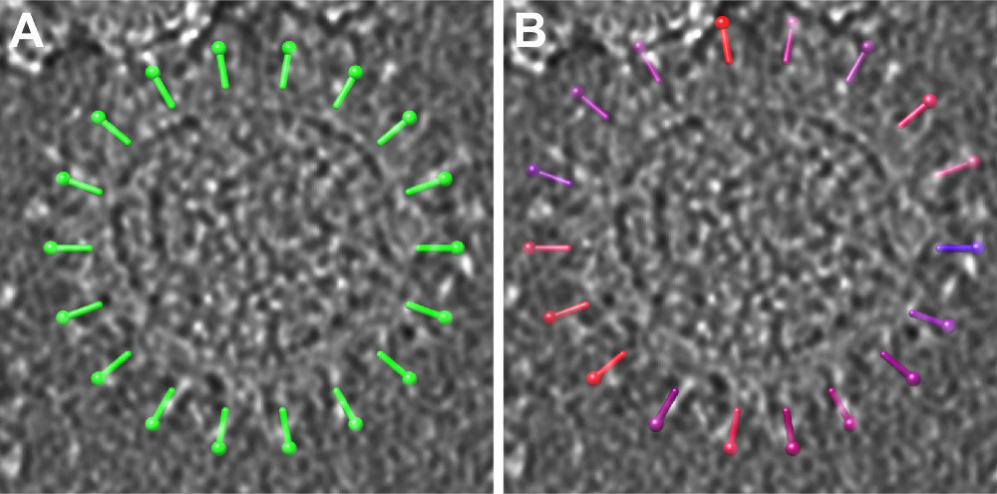
**Figure 4: Refinement of the seeds.A-B)** A subset of seeds before (A) and after (B) refinement are shown on one virion density from **Figure 2**. Seeds in (B) have been color-coded based on the respective cross-correlation coefficients (blue, low correlation; red, high correlation).

The best correlating glycoprotein spike patches (top 75% after removing overlaps; ~1,000 spikes) were used to calculate the final average. The average was resolved to 35-Å resolution (**Figure 5**). It revealed a trimeric spike structure in the middle, in addition to some contribution from six neighboring spikes. Composite models of the virions, calculated by placing the structure in the known positions, revealed placement of the spikes on the virion surface (**Figure 6A**). Occasionally, locally ordered patches of spikes were evident (**Figure 6B**).


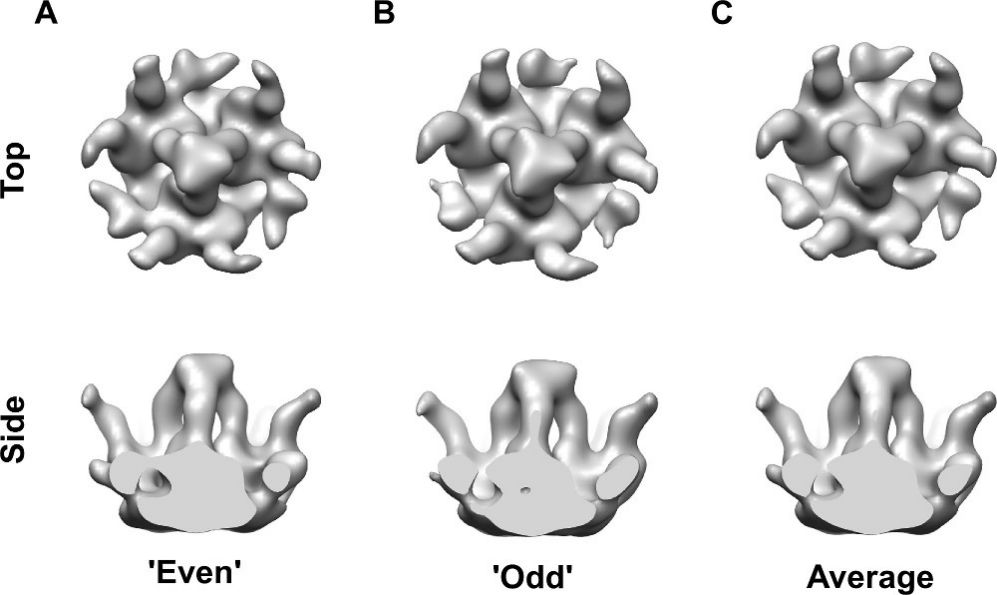
**Figure 5: Refined structure of a patch of the glycoprotein spike layer after template matching. (A-B)** Two maps ‘even’ and ‘odd’, reconstructed from two halves of the data are shown. The two maps show a remarkable degree of similarity, verifying the validness of the approach. The orientation around the spike long axis is different as the two maps have been reconstructed fully independently from each other. (C) Average of the two maps is shown at the final resolution of 35 Å.


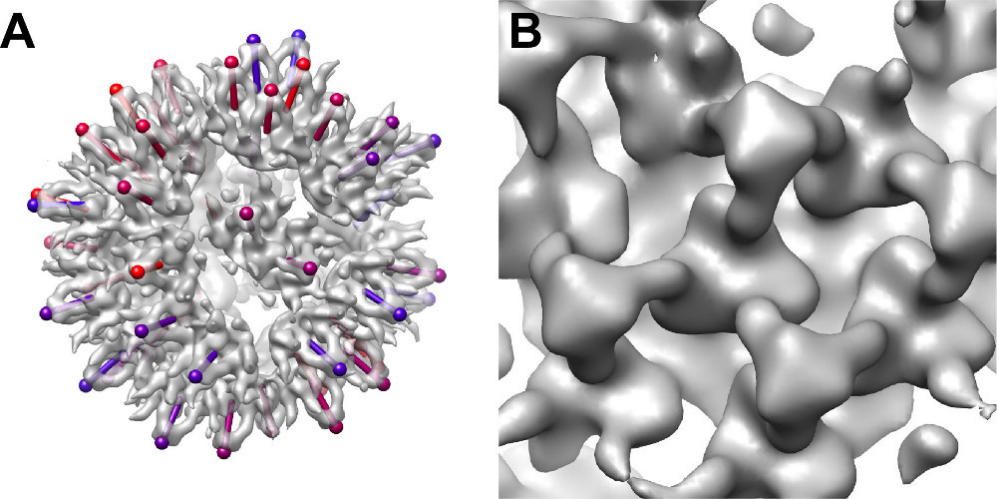
**Figure 6: Placement of the spikes on the Bunyamwera virion. (A)** Composite model of a virion. View vectors (sticks) indicate the orientations of the spikes. Colors indicate the cross-correlation between each spike and the template structure (blue, low correlation; red, high correlation). **(B)** A close-up of an ordered patch of spikes.

## Discussion

Knowledge of viral glycoprotein spike structure on the virion membrane is essential for understanding virus replication and developing therapeutics to treat and prevent infection. Electron cryo-microscopy combined with single particle averaging has emerged as the most utilized method to solve the structures of enveloped virus particles, including the glycoprotein spikes. However, this method is limited to icosahedrally symmetric viruses. Here, through the application of electron cryo-tomography and subtomogram averaging in Jsubtomo, we have outlined a general protocol for determining glycoprotein spikes on pleomorphic enveloped viruses that are not amenable to other current structural biology methods. Our representative results demonstrate that the resolution of this method is sufficient to reveal insights into domain architecture, oligomerization, and higher order organization of glycoprotein spikes on intact virions.

The most critical step within this protocol is constructing two reliable starting models that are statistically independent from one another. Successful execution of this step assumes that glycoprotein spikes are sufficiently large and not packed against each other too tightly, so that individual spikes can be visually recognized and manually picked in the tomograms, and two independent models averaged. If this is not feasible, two modifications to the protocol can be attempted. First, two independent random models can be constructed by first defining two random subsets of subtomograms and then averaging the subtomograms within these subsets^30^. Second, if a structure of the isolated spike has been derived by other means, for example by X-ray crystallography, it can be used as a starting model. However, care must be taken to low-pass filter this model using a low-resolution cut-off (50-70 Å), as the two resulting models in the next round of refinement will be statistically independent only beyond this resolution. Due to this caveat, the former approach is recommended.

The obtainable resolution from this protocol depends on four major factors: i. data collection strategy and the quality of the input data, ii. number of the subtomograms, iii. alignment accuracy of the subtomograms, and iv. heterogeneity of the structures. While the first and second limitation can be largely overcome by using high signal-to-noise direct electron detectors combined with CTF corrected tomography and automated data collection, the alignment accuracy is further affected by the size and shape of the structure of interest itself. When applying this protocol on small spikes lacking prominent features, it may be advantageous to bind Fab fragments to the spike to improve the alignment accuracy and thus resolution^31^. Finally, if the structures to be averaged exhibit multiple conformations, sub-tomogram classification methods may be used to average different conformations separately. To that end, Jsubtomo integrates with the Dynamo package, offering powerful subtomogram classification^9^.

The above protocol is complementary to X-ray crystallography of isolated viral glycoproteins. Crystallographic structures can be fitted into sub-tomogram averages to obtain the precise orientation of the glycoprotein with respect to the virion membrane. Application of this methodology will undoubtedly continue to shed light onto enveloped virus structure and pathobiology.

## Disclosures

The authors have nothing to disclose.

## References

[B0] Nickell S, Mihalache O, Beck F, Hegerl R, Korinek A, Baumeister W (2007). Structural analysis of the 26S proteasome by cryoelectron tomography. Biochemical and biophysical research communications.

[B1] Goldie KN, Wedig T, Mitra AK, Aebi U, Herrmann H, Hoenger A (2007). Dissecting the 3-D structure of vimentin intermediate filaments by cryo-electron tomography. Journal of structural biology.

[B2] Cheng Y, Boll W, Kirchhausen T, Harrison SC, Walz T (2007). Cryo-electron tomography of clathrin-coated vesicles: structural implications for coat assembly. Journal of molecular biology.

[B3] Grünewald K (2003). Three-dimensional structure of herpes simplex virus from cryo-electron tomography. Science.

[B4] Murphy GE, Leadbetter JR, Jensen GJ (2006). In situ structure of the complete Treponema primitia flagellar motor. Nature.

[B5] Medalia O, Weber I, Frangakis A, Nicastro D, Gerisch G, Baumeister W (2002). Macromolecular architecture in eukaryotic cells visualized by cryoelectron tomography. Science.

[B6] Heymann JB, Cardone G, Winkler DC, Steven AC (2008). Computational resources for cryo-electron tomography in Bsoft. Journal of structural biology.

[B7] Kremer J, Mastronarde D, McIntosh J (1996). Computer visualization of three-dimensional image data using IMOD. Journal of structural biology.

[B8] Castaño-Díez D, Kudryashev M, Arheit M, Stahlberg H (2012). Dynamo: a flexible, user-friendly development tool for subtomogram averaging of cryo-EM data in high-performance computing environments. Journal of structural biology.

[B9] Hrabe T, Chen Y, Pfeffer S, Cuellar LK, Mangold A-V, Förster F (2012). PyTom: a python-based toolbox for localization of macromolecules in cryo-electron tomograms and subtomogram analysis. Journal of structural biology.

[B10] Fernández JJ (2012). Computational methods for electron tomography.

[B11] Briggs JAG (2013). Structural biology in situ--the potential of subtomogram averaging). Current opinion in structural biology.

[B12] Frangakis AS (2002). Identification of macromolecular complexes in cryoelectron tomograms of phantom cells. Proceedings of the National Academy of Sciences of the United States of America.

[B13] Zanetti G, Briggs JAG, Grünewald K, Sattentau QJ, Fuller SD (2006). Cryo-electron tomographic structure of an immunodeficiency virus envelope complex in situ. PLoS pathogens.

[B14] Liu J, Bartesaghi A, Borgnia MJ, Sapiro G, Subramaniam S (2008). Molecular architecture of native HIV-1 gp120 trimers. Nature.

[B15] Meyerson JR (2011). Determination of molecular structures of HIV envelope glycoproteins using cryo-electron tomography and automated sub-tomogram averaging. Journal of visualized experiments : JoVE.

[B16] Baker TS, Olson NH, Fuller SD (1999). Adding the third dimension to virus life cycles: three-dimensional reconstruction of icosahedral viruses from cryo-electron micrographs. Microbiology and molecular biology reviews : MMBR.

[B17] Huiskonen JT, Butcher SJ (2007). Membrane-containing viruses with icosahedrally symmetric capsids. Current opinion in structural biology.

[B18] Liljeroos L, Huiskonen JT, Ora A, Susi P, Butcher SJ (2011). Electron cryotomography of measles virus reveals how matrix protein coats the ribonucleocapsid within intact virions. Proceedings of the National Academy of Sciences of the United States of America.

[B19] Arranz R (2012). The structure of native influenza virion ribonucleoproteins. Science.

[B20] Karotki L (2011). Eisosome proteins assemble into a membrane scaffold. Journal of Cell Biology.

[B21] Pietilä MK (2012). Virion architecture unifies globally distributed pleolipoviruses infecting halophilic archaea. Journal of virology.

[B22] Huiskonen JT (2010). Electron cryotomography of Tula hantavirus suggests a unique assembly paradigm for enveloped viruses. Journal of virology.

[B23] Bowden TA, Bitto D, McLees A, Yeromonahos C, Elliott RM, Huiskonen JT (2013). Orthobunyavirus ultrastructure and the curious tripodal glycoprotein spike. PLoS pathogens.

[B24] Maurer UE (1993). The Structure of Herpesvirus Fusion Glycoprotein B-Bilayer Complex Reveals the Protein-Membrane and Lateral Protein-Protein Interaction. Structure.

[B25] Gan L, Ladinsky MS, Jensen GJ (2013). Chromatin in a marine picoeukaryote is a disordered assemblage of nucleosomes. Chromosoma.

[B26] Scheres SHW, Chen S (2012). Prevention of overfitting in cryo-EM structure determination. Nature.

[B27] Heymann JB, Belnap DM (2007). Bsoft: image processing and molecular modeling for electron microscopy. Journal of structural biology.

[B28] Goddard TD, Huang CC, Ferrin TE (2007). Visualizing density maps with UCSF Chimera. Journal of structural biology.

[B29] Faini M (2012). The structures of COPI-coated vesicles reveal alternate coatomer conformations and interactions. Science.

[B30] Wu S (2012). Fabs enable single particle cryoEM studies of small proteins. Structure.

